# Molecular and therapeutic frontiers in anemia therapy

**DOI:** 10.1172/JCI203401

**Published:** 2026-05-15

**Authors:** Nilesh Rai, Omar Abdel-Wahab, Lingbo Zhang

**Affiliations:** 1 Cold Spring Harbor Laboratory, Cold Spring Harbor, New York, USA.; 2 Molecular Pharmacology Program, and; 3 Leukemia Service, Memorial Sloan Kettering Cancer Center, New York, New York, USA.

## Abstract

Anemia affects one-third of the population globally and is marked by impaired erythropoiesis that results in substantial mortality and morbidity. Over the past few decades, our understanding of the molecular mechanisms underlying anemia has progressed but translating that knowledge into effective targeted therapeutics remains challenging. Preclinical and clinical studies substantiate the efficacy of modulating erythropoietin-driven signaling pathways to stimulate erythropoiesis. Additional approaches include strategies to maintain iron homeostasis and control iron metabolism, using small molecules and oral supplements. New frontiers in molecular regulation of anemia include perturbation of regulatory genes and spliceosome proteins in erythroid cells, as well as mutation-specific therapeutic approaches. Finally, new evidence supporting the importance of neuronal signaling and mitochondrial dynamics in shaping erythropoiesis is pointing toward novel interventions. Here, we discuss the molecular and genetic factors underlying defective erythropoiesis and highlight current and emerging therapies, including molecular targets to overcome drug resistance.

## Introduction

Anemia affects human health worldwide and is characterized by impaired erythropoiesis or low hemoglobin levels. Anemia can be broadly classified as inherited or acquired (1–3). Inherited forms result from germline defects affecting erythropoiesis and hemoglobin, and include β-thalassemia, α-thalassemia, Fanconi anemia, Diamond-Blackfan anemia, and sickle cell disease (SCD). Acquired forms arise after birth due to blood loss or nutritional insufficiency and include iron deficiency anemia and pernicious anemia. Besides these forms, disorders of bone marrow failure, such as myelodysplastic syndromes (MDS), cause anemia characterized by ineffective erythropoiesis. Despite these differing underlying causes, anemia disease mechanisms converge on defects in erythroid differentiation, survival, or maturation resulting in impaired erythropoiesis (4, 5). In the past few decades, advances in understanding the genetic defects underlying hematological diseases have enabled the translation of several targeted therapeutic approaches for anemia (Table 1). However, currently available treatments still have various limitations, and treatment resistance remains a major unmet clinical challenge, highlighting the need for new therapies that target molecular pathways involved in erythropoiesis.

Erythropoiesis is orchestrated by an interconnected network of signaling molecules, posttranscriptional mechanisms, systemic feedback loops, and metabolic processes that work together to guide erythroid progenitor differentiation (6, 7). During embryogenesis, the aorta-gonad-mesonephros region, a major intra-embryonic site, begins producing hematopoietic stem cells (HSCs), which subsequently colonize the fetal liver. At birth, fetal liver–derived HSCs translocate to the specialized microenvironment of bone marrow within erythroblastic islands (8). Those HSCs give rise to multipotent progenitors, followed by megakaryocyte/erythroid progenitors that progress through an ordered sequence of erythroid stages, initially forming as burst-forming unit-erythroid (BFU-E) progenitors, which then transition into colony-forming unit-erythroid (CFU-E) progenitors. CFU-Es gradually differentiate into proerythroblasts, then basophilic, polychromatic, and orthochromatic erythroblasts (9–11). The orthochromatic erythroblasts undergo chromatin condensation and enucleation to produce reticulocytes that become fully functional red blood cells (RBCs) (12–16). An abnormality at any stage of erythropoiesis — caused by changes in erythroid-specific genes, genetic mutations, or chromosomal abnormalities — can cause anemia.

Although substantial advances have been made in our understanding of erythropoiesis, translating existing knowledge into effective molecular or gene-based therapeutics for anemia remains challenging. Here, we focus on the intricate molecular and cellular regulation of erythropoiesis and its dysregulation in various anemia-associated disorders. We discuss the signaling pathways, splicing machinery, genetic regulators, and metabolic processes that regulate erythropoiesis. We highlight innovative therapeutic approaches, including FDA-approved clinical interventions and preclinical practices, integrating advances in genomics and CRISPR-based gene editing. Furthermore, we emphasize potential novel therapeutic strategies for anemia, focusing on iron metabolism, neuronal components, and mitochondrial dynamics. Overall, a detailed understanding of erythropoietic mechanisms is necessary to achieve our unmet potential for targeted therapies focused on correcting the core etiologies of anemia.

## Transduction signaling molecules regulate erythropoiesis

**Figure 1:**
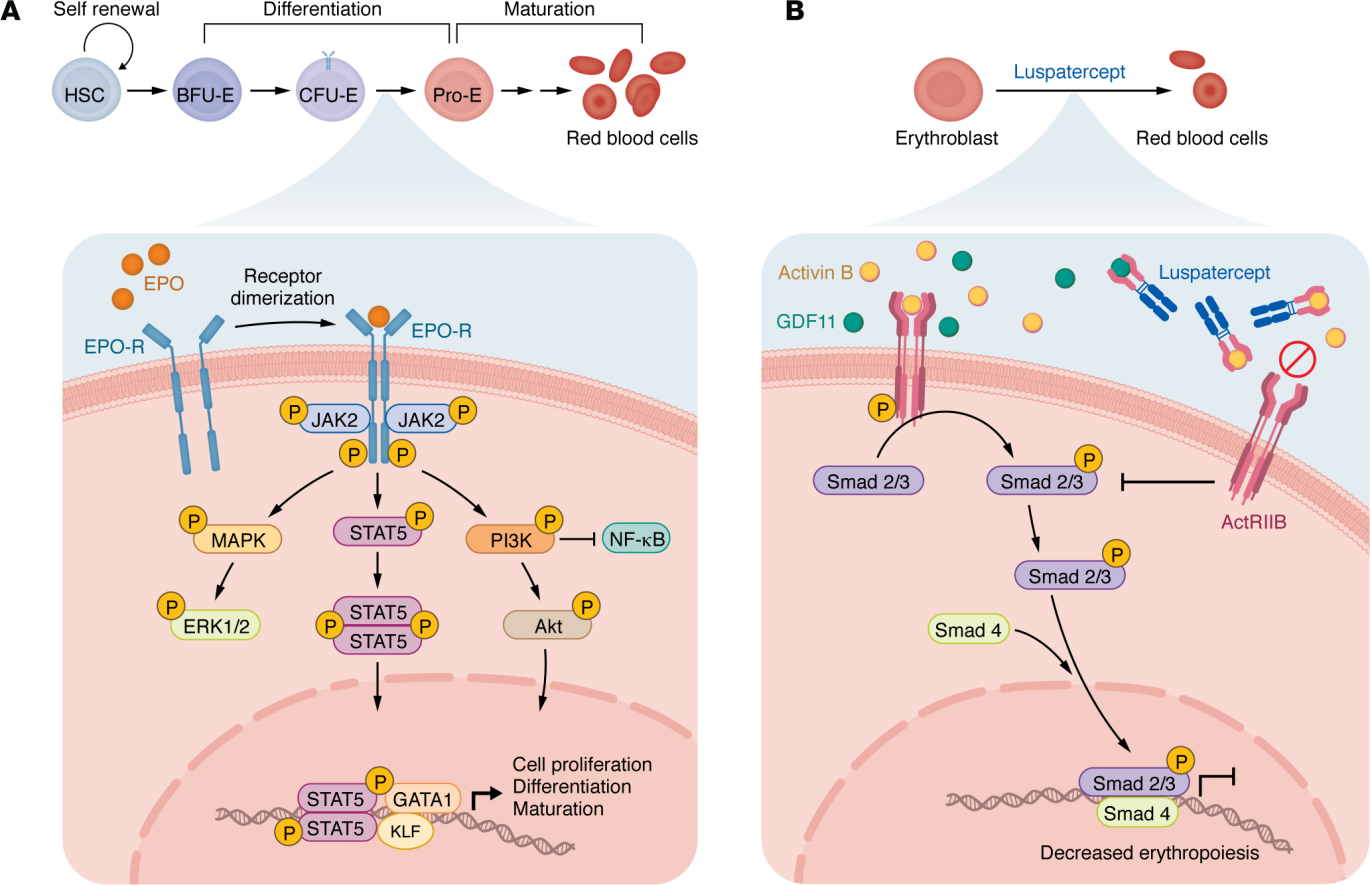
Signal transduction–based therapies for anemia. (**A**) Erythropoietin-mediated (EPO-mediated) key signaling pathways regulate erythroid progenitor differentiation and maturation. EPO interacts with its receptor (EPO-R), phosphorylating JAK2 and activating downstream STAT5, MAPK, and PI3K/Akt transduction pathways, which promote cell division, maturation, and proliferation. KLF, Krüppel-like factor. (**B**) Luspatercept-mediated inhibition of the SMAD2/3 pathway. Luspatercept traps ligands such as activin B and GDF11, inhibiting their interaction with activin receptor type IIB. This prevents the phosphorylation of Smad2/3 and their binding to the coactivator Smad4, thereby activating late-stage erythroid-specific genes and promoting erythropoiesis by inhibiting Smad2/3 signaling in transfusion-dependent anemia. ActRIIB, activin receptor type IIB; GDF11, growth differentiation factor 11.

Erythropoiesis is regulated by major transduction signaling molecules, principally erythropoietin (EPO) and its receptor (EPO-R). EPO stimulates canonical JAK2/STAT5, PI3K/Akt, and MAPK pathways, which are indispensable for facilitating erythroid progenitor survival and differentiation (Figure 1A). In humans, EPO deficiency or defects in EPO-R signaling impair the survival and differentiation of erythroid progenitors, while their targeted deletion in mice causes death on embryonic day 13, indicating the importance of this signaling pathway in erythropoiesis (17–19). Any aberration in signaling downstream of EPO-R affects erythroid cell differentiation and maturation, culminating in anemia. Here, we discuss signal transduction–based anemia therapeutics, including EPO, erythropoiesis-stimulating agents (ESAs), and luspatercept.

### Key signaling pathways during erythropoiesis.

Following EPO binding, EPO-R homodimerizes and undergoes conformational changes that facilitate JAK2 activation (20). JAK2/STAT5 signaling regulates genes responsible for erythroid expansion and differentiation. Phosphorylation of STAT5 fosters the transcription of erythroid-specific and antiapoptotic genes, thereby promoting growth and survival of progenitors. Parallel to this, EPO-mediated JAK2/STAT5 signaling facilitates tyrosine 479 (Tyr479) phosphorylation at the cytoplasmic end of EPO-R, where PI3K binds through its regulatory subunit p85 (21, 22). Moreover, phosphorylation of Grb-associated binder (Gab) and insulin receptor substrate-2 (IRS-2) adaptor proteins are two additional independent mechanisms for EPO-dependent PI3K activation in erythroid cells (23). Akt, the downstream serine/threonine kinase of PI3K, phosphorylates GATA1 (at conserved serine 310) and enhances erythroid differentiation and maturation (24, 25). In concert with these mechanisms, EPO/EPO-R signaling and stem cell factor (SCF) signaling via c-Kit also initiate the RAS/MAPK signaling cascade, which promotes proliferation of erythroid progenitors and is gradually inhibited as differentiation progresses. EPO-induced proliferation is suppressed by Raf-1 inhibition, demonstrating the importance of Ras/ERK signaling in erythropoiesis (26). Similarly, inhibition of PI3K activity impairs differentiation and maturation of primary erythroid progenitors (27). Conversely, inhibition of caspase-mediated GATA1 degradation by activated HSP70 improves erythroid cell survival, promoting healthy erythropoiesis (28). Taken together, these studies indicate that JAK2/STAT5, PI3K/Akt, and MAPK transduction pathways are all crucial for healthy erythropoiesis.

### Therapeutic targeting of transducing molecules.

Given its central role in erythropoiesis, treatment with EPO itself is a logical approach to anemia therapy. EPO is a 165–amino acid pleiotropic hormone produced by peritubular cells under hypoxic conditions. Initially, clinical studies using native, non-recombinant EPO demonstrated its marked efficacy in treating anemia and ineffective erythropoiesis (29–31). Later, forms of recombinant human EPO (rhEPO), often called ESAs, were developed by cloning the human *EPO* gene (32, 33). The first ESA, epoetin alfa, engages EPO-R on erythroid progenitor cells and triggers RBC production (34). Other notable ESAs, such as continuous erythropoietin receptor activator (CERA) and darbepoetin-α, were engineered to have extended half-lives for anemia therapy (35–37). The unique pharmacokinetic and receptor-binding characteristics of CERA make it distinct from currently available ESAs (38). An alternative to this approach is the chimeric activator 10F7-EPO^R150A^, which was engineered by coupling the RBC-specific glycophorin A–binding antibody 10F7 to mutated EPO (EPO^R150A^). 10F7-EPO^R150A^ has reduced affinity for EPO-R to facilitate targeted erythropoiesis while avoiding thrombosis (39). Notably, in addition to its function in regulating erythropoiesis, EPO has been reported to regulate multiple physiological functions in non-erythroid tissues, with ongoing research exploring its roles in the brain and kidneys (40–42).

In MDS, active TGF-β/SMAD signaling leads to ineffective erythropoiesis by mitigating terminal differentiation (43). Therefore, beyond EPO-based treatments, recent advances have focused on targeting the TGF-β/SMAD pathway to restore erythroid differentiation. In response to TGF-β ligands binding their receptors, SMAD2 and SMAD3 assemble into a heteromeric complex with SMAD4 and subsequently shuttle to the nucleus to regulate erythroid genes. Luspatercept is a recombinant activin receptor novel fusion protein that blocks SMAD2/SMAD3 signaling by trapping ligands of the TGF-β family, like activin B and growth differentiation factor 11 (GDF11) (Figure 1B) (44). In phase II and III clinical studies, luspatercept was shown to enhance hemoglobin levels in comparison with placebo among lower-risk MDS and transfusion-dependent patients (45–47). It was subsequently approved for clinical use by the FDA (45, 48, 49).

## Targeting metabolism of iron and its receptors

In healthy humans, total body iron stores are approximately 3–4 grams (50), and this is required for hemoglobin production and oxygen transport. The liver primarily regulates iron balance through a tightly controlled iron cycle that manages dietary absorption, plasma transport, cellular use, storage, and macrophage recycling. Dysregulation of iron homeostasis can cause anemia, either due to reduced iron levels or iron overload (51, 52). Here, we discuss changes in iron balance and its regulation in anemia therapeutics.

### Iron homeostasis in erythropoiesis.

Dietary iron absorption is the primary route by which iron enters the systemic circulation. The absorption of iron from food is facilitated by divalent metal transporter 1 (DMT1) in the duodenum (53). Within enterocytes, iron is either sequestered as ferritin or exported via the iron transporter ferroportin (Fpn) (54). Exported ferrous iron is oxidized to ferric iron and binds to transferrin in plasma, ensuring effective transport to peripheral tissue. Circulating transferrin-bound iron serves as the main iron source that promotes healthy erythropoiesis. Transferrin is a high-affinity receptor–mediated transport system recognized by TfR1 on erythroblasts, thereby potentiating iron uptake to support hemoglobin synthesis and erythropoiesis (55, 56). At the cellular level, clathrin-mediated endocytosis internalizes iron-containing transferrin after TfR1 interaction. Iron is liberated inside the acidic endosome, while apotransferrin (iron-free transferrin) and TfR1 get transported back to the plasma membrane and liberated into the bloodstream (57). Under transferrin-saturated or iron overload conditions, excess plasma non–transferrin-bound iron is imported via transporters, such as ZIP8 and ZIP14, especially in hepatocytes and other parenchymal tissues (58, 59).

The iron overload toxicity is prevented, and adequate iron intake for erythropoiesis is ensured, by regulating iron homeostasis (60). Splenic macrophages break down senescent erythrocytes with the support of liver and bone marrow to liberate iron from heme for reuse in hemoglobin synthesis (61, 62). During this process, hepatocyte-produced hepcidin regulates recycling of iron in circulation by directly associating with Fpn, thereby triggering its internalization and lysosomal degradation. Surplus iron that is not immediately required for erythropoiesis is accumulated in hepatocytes or in enterocytes as ferritin. Additionally, the synergistic activity of hephaestin and ceruloplasmin, two multicopper ferroxidases, promotes Fe³^+^ loading onto plasma transferrin and iron efflux, linking copper metabolism to iron deficiency anemia (63, 64).

Iron imbalances can lead to anemia when there is insufficient iron to produce the hemoglobin necessary for erythrocytes (51, 65). Iron deficiency initially reflects an early decline in total-body iron stores, occurring before the development of frank anemia. Iron deficiency anemia is a severe pathological condition characterized by depleted iron stores, microcytic, hypochromic RBCs, and diminished oxygen transport. These pathological conditions can be caused by changes in iron metabolism driven by inflammation, dysregulated hepcidin signaling, changes in TfR expression, and impaired ferritin storage ability (66). Moreover, commonly consumed pharmaceuticals, including NSAIDs and anticoagulants, may cause blood loss resulting in anemia, and proton-pump inhibitors can impede iron absorption (67).

### Targeting iron homeostasis in anemia.

Iron homeostasis is crucial for erythropoiesis, and its disruption leads to several types of anemia, including iron deficiency, β-thalassemia, and chronic kidney disease–associated anemia (68, 69). Iron-related anemia is treated with various therapeutic strategies aimed at regulating hepcidin levels, enhancing iron absorption, and managing iron overload (Table 2). Clinically approved treatments focus on iron replacement or chelation and remain the standard of care for iron deficiency and overload anemia. Emerging therapeutic approaches aim to regulate iron sensing and distribution through the hepcidin/ferroportin axis and its upstream regulators, including BMP6, TMPRSS6, and erythroferrone. Transferrin plays a central role in iron delivery to bone marrow, even though it carries only 0.1% of the total body iron. Transferrin is a potent regulator of inefficient erythropoiesis, due to its rapid turnover and preferential targeting of erythropoietic tissues. In a mouse model of β-thalassemia, transferrin treatment increases hemoglobin and hematocrit while decreasing reticulocyte counts, EPO levels, and splenomegaly. Early on, transferrin treatment mobilizes iron from the liver and heart, whereas extended treatment (~60 days) reduces iron overload by mobilizing iron from the spleen (70).

Beyond iron transport, iron homeostasis is primarily controlled by hepcidin, which lowers plasma iron levels by promoting Fpn internalization and degradation (71). TMPRSS6 negatively regulates the canonical BMP pathway and reduces expression of the gene encoding hepcidin (*HAMP*) (72–74). Accordingly, TMPRSS6 inhibitors, hepcidin modulators, and hepcidin reduce iron overload and manage iron homeostasis. Minihepcidins, synthetic hepcidin agonists, alleviate anemia and excessive iron levels in β-thalassemic mice (Hbb^th3/+^). Interestingly, treatment with minihepcidin reduces iron overload by preventing new iron entry into the system, whereas iron-chelating agents, such as deferoxamine (DFO), decrease the existing iron burden (75). Overall, these findings demonstrate that targeting iron transport, absorption, and regulatory molecules offers multiple approaches to alleviate iron-related anemias.

## Genetic regulation in erythropoiesis

Genetic networks regulate erythroid survival, differentiation, and hemoglobinization throughout erythropoiesis by coordinating erythroid gene expression (4, 76). Disruption of these networks through changes in transcription factors and *cis*-regulatory elements can impair erythroid differentiation, alter globin gene expression, and reduce erythrocyte survival, resulting in inherited anemia. For example, SCD results from the substitution of a single nucleotide in the β-globin gene that causes the production of sickle hemoglobin (HbS), leading to erythrocyte sickling and chronic hemolysis (77).

Fetal hemoglobin (HbF) must be adequately present and evenly distributed within erythrocytes to effectively prevent HbS polymerization (78). HbF expression is controlled by a well-coordinated transcriptional network that orchestrates the switch from ε-globin to γ-globin during the early developmental phase and the switch from γ-globin to β-globin at birth. Importantly, transcription factors such as BCL11A, ZBTB7A, and GATA1 collectively regulate γ-globin silencing during erythropoiesis (79). These factors function through tightly arranged *cis*-regulatory elements within the γ-globin promoter, including proximal NF-Y–binding CCAAT boxes and distal BCL11A-binding motifs, along with upstream GATA1 and ZBTB7A motifs (80). Repressors and activators compete for binding sites during γ-globin regulation, exerting antagonistic effects. Furthermore, this competition is driven by steric interference, which can prevent the simultaneous binding of ZBTB7A and GATA1 to adjacent motifs. Likewise, BCL11A binds to the tandem motif and recruits the NuRD complex corepressor, which attenuates NF-Y–mediated activation of γ-globin in erythroid cells (81, 82). Disruption of this regulatory system in erythroid cells, including the loss of BCL11A function, changes in its promoter binding site, or hereditary persistence of HbF, shifts gene regulation toward γ-globin expression. These changes facilitate GATA1 binding and reduce transcriptional repression, highlighting the cross-regulation between the activator and repressor motifs (83). Together, these observations indicate that mutations affecting globin regulators or their DNA-binding sites disrupt normal erythroid maturation, alter HbF levels, and impair erythropoiesis, thereby influencing the severity of SCD. Overall, globin gene regulation reflects genetic control mechanisms in erythropoiesis and may affect transcriptional networks relevant to anemia treatment.

### Genetic approaches for correcting anemia.

**Figure 2:**
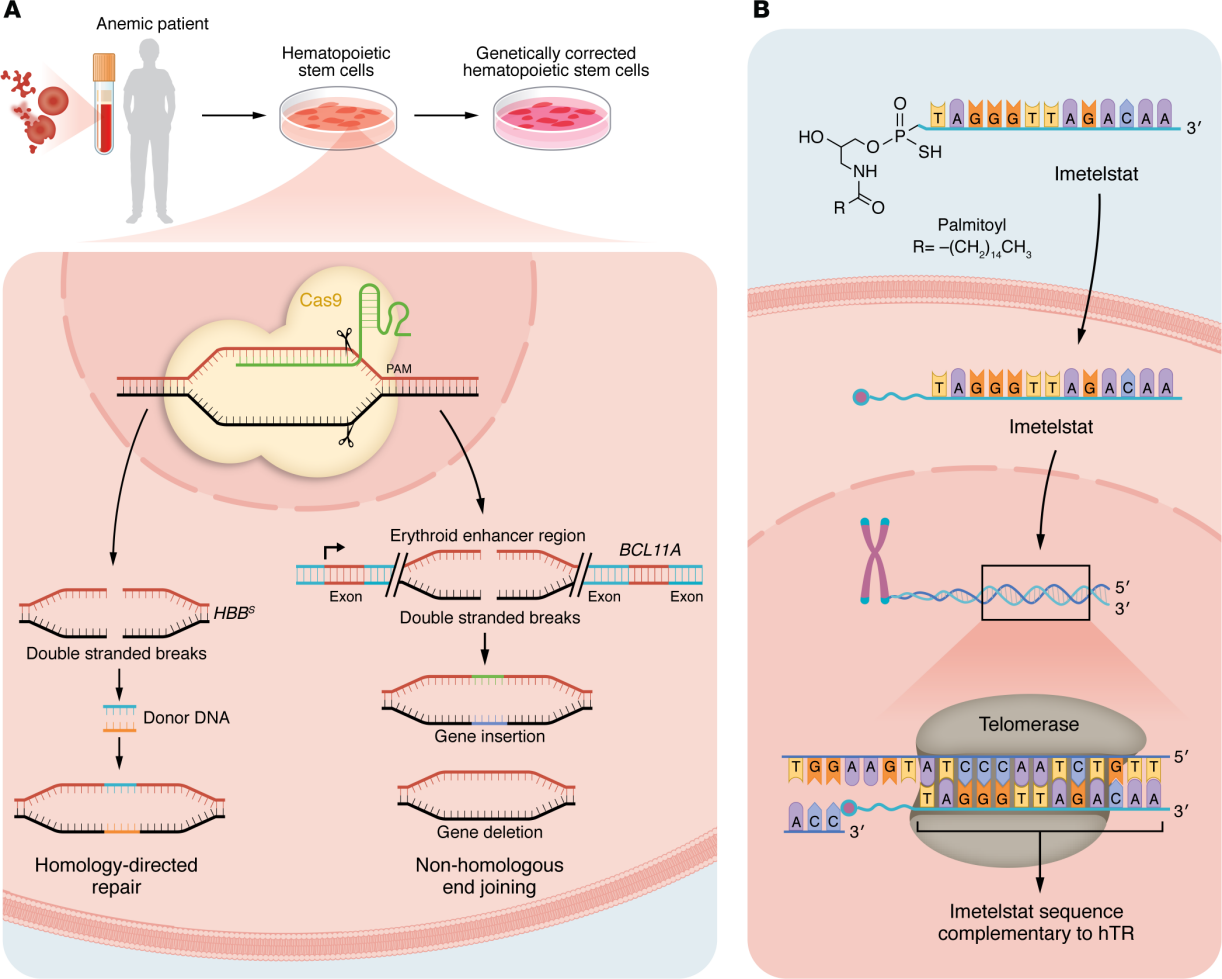
Genetic strategies for the treatment of anemia. (**A**) Genetic tools for genome editing of erythroid regulators. HSCs are isolated and double-stranded breaks induced using CRISPR/Cas9, followed by NHEJ and HDR. HDR-based correction of the βS mutation uses adeno-associated virus 6–delivered donor DNA to replace the mutant codon with the wild-type and restore normal adult hemoglobin (HbA). The disruption of the erythroid-specific *BCL11A* enhancer region through the NHEJ-mediated indel formation decreases BCL11A expression and reactivates HbF in erythroid cells. PAM, protospacer-adjacent motif. (**B**) Mechanism of action of imetelstat. The lipid moiety at the 5′ end of the 13-base nucleotide sequence facilitates cellular uptake of imetelstat and its binding to the complementary human telomerase repeat (hTR) sequence. Following binding, imetelstat competitively inhibits the telomerase activity in malignant HSPCs in low-risk MDS.

In the realm of anemia therapeutics, efforts to control HbF gene expression encompass pharmacologic compounds and cell-based therapies, and viral vector–based approaches now also enable CRISPR/Cas9-mediated gene correction in autologous HSCs (84–87). GWAS-based genomic studies have identified several quantitative trait loci that regulate HbF expression, including the *BCL11A* promoter. The regulatory landscape of *BCL11A* includes functional variants marked by SNPs within intron 2, which harbors an erythroid-specific enhancer (83, 88). Recently, the FDA approved Casgevy (also referred to as exagamglogene autotemcel), which targets the erythroid-specific enhancer region in *BCL11A*, as a first-line CRISPR/Cas9-based therapy for β-thalassemia and SCD (89, 90). In this approach, CRISPR/Cas9 introduces targeted double-strand breaks in *BCL11A* that are repaired by non-homologous end joining (NHEJ) resulting in an indel, enabling efficient genetic modification of the *BCL11A* erythroid-specific enhancer region (Figure 2A). The NHEJ-driven approach suppresses BCL11A, the primary inhibitor of γ-globin genes (*HBG1* and *HBG2*), thereby reactivating HbF, which blocks HbS polymerization and hemolysis and reduces RBC sickling. In contrast, the investigational therapy GPH101 uses CRISPR/Cas9 and homology-directed repair (HDR) to correct the *HBB* mutation ex vivo via an adeno-associated virus 6 containing a delivery template (91). GPH101-mediated correction promotes the production of HbA and decreases HbS. This approach resulted in genetically engineered HSCs that differentiated into erythroid cells that generated a minimum of 70% HbA.

In addition, several FDA-approved drugs have attracted attention for their targeted mechanisms in treating SCD (86). Voxelotor increases the affinity of hemoglobin for oxygen to prevent deoxy-HbS polymerization, while crizanlizumab inhibits P-selectin–mediated adhesion of leukocytes and sickled erythrocytes to the endothelium (92–94). L-Glutamine targets downstream pathophysiological pathways, including oxidative stress, vaso-occlusive crisis, and hemolysis (95). Hydroxyurea, an FDA-approved HbF-inducing therapy, remains the standard of care; however, adult patients with SCD rarely achieve the high HbF levels seen in young children, which are crucial for improving disease outcomes (96).

Recently, a genetics-based approach was developed to treat anemia associated with MDS. In clinical trials, imetelstat, an FDA-approved 13-base oligonucleotide containing a 5′-end lipid moiety, has been shown to alleviate transfusion-dependent anemia (97–101). Imetelstat inhibits the reverse transcriptase telomerase, which is often elevated in MDS, thereby eliminating malignant cells with high telomerase activity (Figure 2B). The phase III IMerge trial included 178 lower-risk MDS and transfusion-dependent patients, and they were randomly assigned to placebo- or imetelstat-treated groups (99, 101). In contrast with the 15% of patients receiving placebo, 40% patients treated with imetelstat attained RBC transfusion independence for up to 8 weeks. At the same time, 28% of imetelstat-treated patients achieved up to 24-week transfusion independence, compared with 3% of placebo-treated patients (99), although a high rate of cytopenia was observed in these patients.

## Splicing machinery and its importance in erythropoiesis

In eukaryotes, splicing is a well-regulated transcriptional and posttranscriptional mechanism initiated by a stage-specific proteasomal complex, leading to the formation of specialized cell types. During erythropoiesis, proper splicing is required for the production of the 4.1R protein, which stabilizes the erythrocyte membrane (102). Here, we highlight the significance of spliceosome proteins U2 small nuclear RNA auxiliary factor 1 (U2AF1), serine/arginine-rich splicing factor 2 (SRSF2), and splicing factor 3B subunit 1 (SF3B1) in erythroid development and maturation (103), and emphasize splicing-based therapeutics in MDS aimed at restoring erythropoiesis.

### Significance of spliceosomal proteins in erythropoiesis.

**Figure 3:**
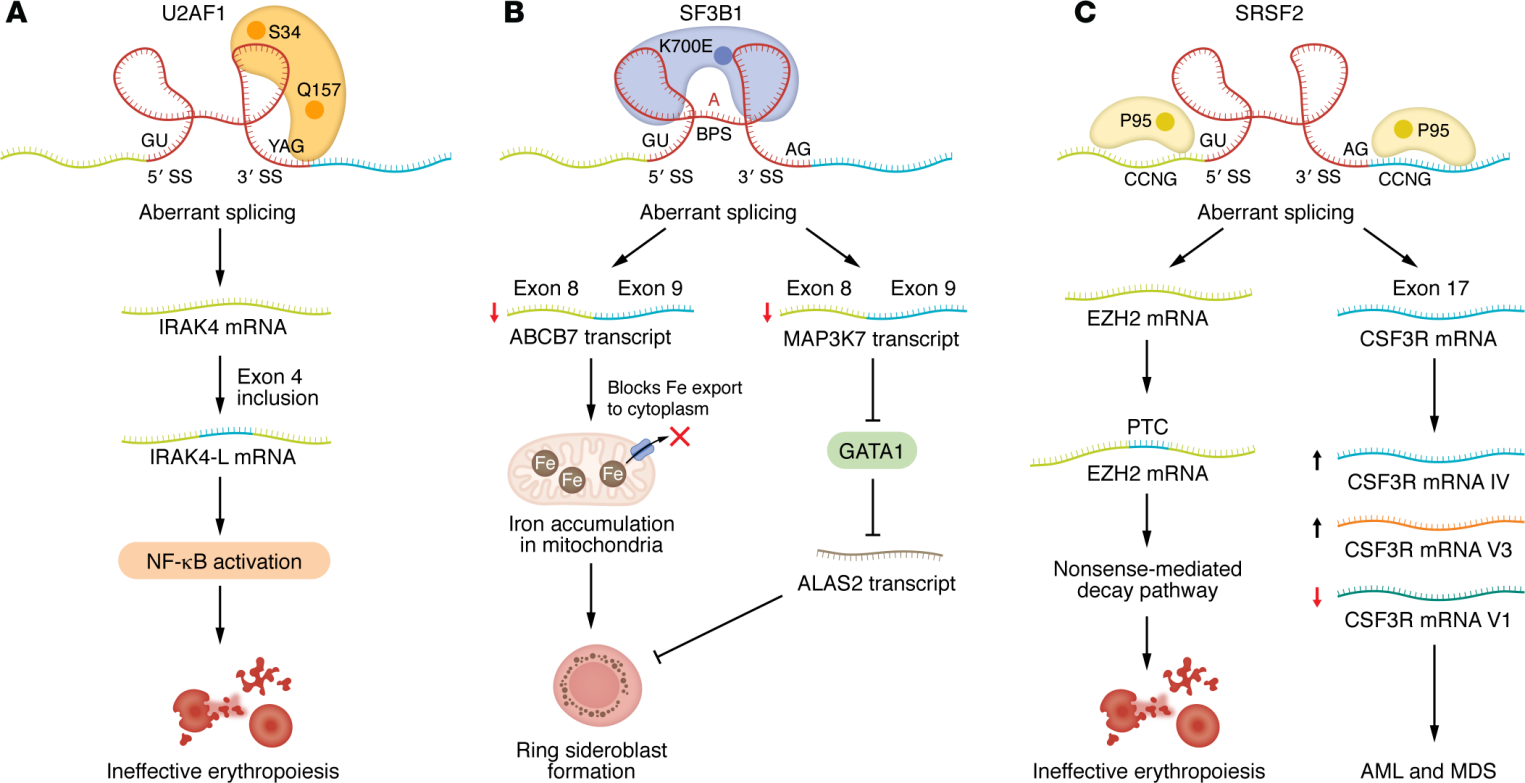
Molecular mechanisms of spliceosome protein mutation–induced ineffective erythropoiesis. (**A**) *U2AF1* mutations cause ineffective erythropoiesis. Hotspot mutations in *U2AF1* (S34 and Q157) trigger aberrant splicing, promoting the accumulation of the IRAK4 active isoform, IRAK4-L, upon inclusion of exon 4. IRAK4-L accumulation activates NF-κB signaling, contributing to ineffective erythropoiesis. (**B**) *SF3B1* mutation promotes ring sideroblast formation in MDS. The mutant SF3B1 protein (SF3B1^K700E^) uses an aberrant branch point site (BPS) and cryptic 3′ splice sites. The resulting aberrant splicing of ABCB7 and MAP3K7 transcripts impairs iron export, leads to mitochondrial iron accumulation, and results in ring sideroblast formation. Disrupted MAP3K7 splicing also impairs GATA1 and ALAS2 expression, further affecting erythroid maturation and promoting ring sideroblast formation. (**C**) *SRSF2* mutation alters splicing and triggers ineffective erythropoiesis. The *SRSF2*^P95H^ mutation promotes mis-splicing of EZH2 mRNA by introducing a premature termination codon (PTC). Subsequently, EZH2 mRNA is degraded via nonsense-mediated decay, resulting in ineffective erythropoiesis. In MDS, SRSF2^P95H^ binds the CCNG motif and induces the aberrant class IV splicing of colony-stimulating factor 3 receptor (CSF3R) mRNA. V3 and V1 are two spliced variants of CSF3R. V1 is the full-length, signaling-competent receptor, while V3 by itself results in a hypoproliferative trait and decreased JAK/STAT activity. In AML, SRSF2^P95H^ also increases the CSF3R variant 3/1 (V3/V1) ratio, leading to leukemogenic transformation.

Spliceosomes orchestrate alternative splicing by recognizing splice sites within introns and exons to generate functionally distinct protein isoforms that are crucial for erythroid maturation. Meticulous oversight of splicing processes is required for progenitor maturation and the formation of healthy RBCs. U2AF1, SF3B1, and SRSF2 are key splicing factors that participate in a common RNA splicing pathway, and mutations in the genes encoding these factors give rise to various forms of anemia and tumors (103, 104). Specifically, heterozygous mutations in *U2AF1* (Figure 3A), *SF3B1* (Figure 3B), and *SRSF2* (Figure 3C) are prevalent among patients with various hematological diseases, including MDS. Deletion of the long arm of chromosome 5 (Chr5q deletion), a common cause of MDS, is often associated with mutations in genes that govern RNA splicing, specifically *U2AF1* (105). These spliceosome mutations cause prevalent gene expression changes that disrupt erythropoiesis and exacerbate anemia by impairing normal pre-mRNA processing (106). *U2AF1* mutations at residues S34 and Q157 affect its recognition of the 3′ splice site, which promotes inclusion of exon 4 in the IRAK4-L isoform, which activates NF-κB and leads to ineffective erythropoiesis. The *SF3B1* mutation K700E is often specific to MDS subtypes and serves as a diagnostic marker (107, 108). The *SF3B1* mutation causes exon skipping during the splicing of the iron transporter gene *ABCB7* and of *MAP3K7* by affecting branch-point recognition (109, 110). In low-risk MDS with ring sideroblasts (MDS-RS), this alternative splicing of *ABCB7* results in excessive iron deposition in mitochondria and subsequent erythropoiesis impairment (111). *SRSF2* mutations, particularly at codon P95, are more frequent in chronic myelomonocytic leukemia, as SRSF2 depletion leads to genomic instability by altering the recognition of CCNG motifs at 5′ and 3′ splice sites (112, 113). Intriguingly, *SRSF2* mutations have a greater incidence of concurrent gene mutations than *U2AF35* mutations (114–116), highlighting their broader pathological impact. Overall, targeting RNA splicing factors is a promising therapeutic target for anemia, particularly in spliceosome-mutant MDS.

### Splicing-based therapeutic interventions for anemia.

Splicing-modulation-based therapies are exciting prospects for correcting aberrant gene expression patterns in anemic patients. Initial studies focusing on splicing-modulating drugs such as E7107, H3B-8800, and E7820, and antisense oligonucleotides (ASOs) have shown promise for improving erythroid maturation and erythrocyte production (117–119). E7107, a spliceosome regulator, showed greater inhibition of splicing in *SF3B1^K700E^* mutant cells than in non-mutated cells, indicating that spliceosome targeting is a promising approach for managing anemia caused by *SF3B1* mutations (120). Further studies led to the development of an oral SF3B modulator, H3B-8800, which demonstrated efficacy in *SF3B1*-, *SRSF2*-, and *U2AF1*-mutant cells by retaining GC-enriched short introns (121). Interestingly, H3B-8800 tightly binds the SF3B complex and inhibits canonical and peculiar splicing in *SF3B1*-mutant cells. In a phase I clinical study, H3B-8800 showed safety with prolonged dosing and modest clinical efficacy, including substantially improved erythropoiesis and reduced RBC and platelet transfusion in spliceosome-mutant MDS patients (118, 122). Additional anemia therapeutic approaches targeting alternative splicing include the controlled degradation of splicing factors. For example, the oral drug E7820 was developed to promote degradation of the splicing factor RBM39 and tested as anemia therapy in patients with acute myeloid leukemia, MDS, or chronic myelomonocytic leukemia with recurrent spliceosome mutations (e.g., *SF3B1*, *SRSF2*, *U2AF1*) (117, 123). E7820 monotherapy at the maximum tolerated dose exhibited modest efficacy such as transient reduction in marrow blasts, suggesting the need for combinational therapy (124).

In addition to small molecules, ASO-based techniques enhance splicing precision by targeting mis-spliced transcript variants or by regulating exon inclusion in erythroid genes (125). Splice-switching oligonucleotides regulate splicing by blocking the binding of spliceosomal or regulatory factors, thereby altering exon inclusion or exclusion without inducing RNA degradation (126). In a preclinical MDS model, targeting STAT3 with an ethyl-modified ASO promoted erythroid cell differentiation (127). *SRSF2* mutations in MDS are associated with abnormal splicing of downstream targets EZH2 and BCOR, and ASO-based strategies prevented nonsense-mediated degradation of EZH2 transcripts, partially rescuing hematopoietic defects (128, 129). Of note, the clinical potential of oligonucleotide-based techniques to target the spliceosome in MDS mutant cells is limited by several challenges, such as clinical efficacy, immune responses, and tissue-specific off-targeting effects (125, 130).

## Neuronal signaling in BFU-E differentiation

A hallmark of unresponsiveness to ESA and luspatercept is elevated plasma EPO levels, attributed to diminished differentiation of early erythroid progenitor BFU-Es (131, 132). Emerging evidence suggests that BFU-E differentiation is critically mediated by neuronal interactions through the neurotransmitter receptor muscarinic acetylcholine receptor M4 (CHRM4). CHRMs are G protein–coupled receptors (GPCRs) that are crucial for regulating various physiological functions, and CHRM4 is associated with the Gi/Go protein, which prevents the activity of adenylyl cyclase and inhibits downstream pathways by reducing cyclic AMP levels (133).

**Figure 4:**
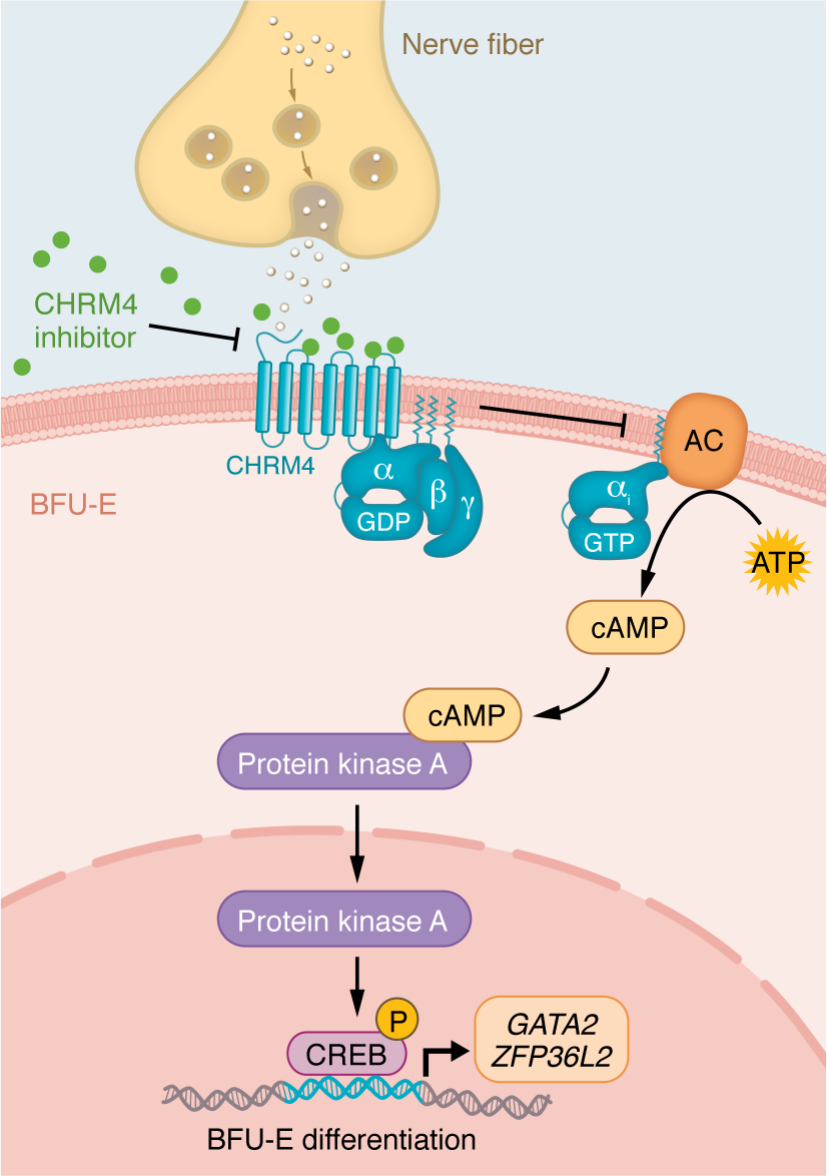
Targeting of neurotransmitter receptor CHRM4 to regulate early erythroid progenitor differentiation. Neurotransmitter receptor CHRM4 is coupled with the Gi/Go protein, which prevents the activation of adenylyl cyclase (AC) and inhibits the G protein–coupled pathway. Inhibition of CHRM4 triggers the elevation of cAMP, which activates protein kinase A (PKA) and phosphorylates the downstream transcription factor CREB. The CREB transcriptional program upregulates target genes GATA2 and ZFP36L2, driving BFU-E differentiation.

In bone marrow, CHRM4 governs the differentiation of BFU-E cells by modulating the cAMP/CREB signaling pathway (Figure 4). In mice, knockout of CHRM4 results in enhanced differentiation of BFU-E cells (134, 135). Inhibition of CHRM4 increases cAMP levels and alters the transcriptional activity of CREB, whereas depletion of CREB reduces the regulatory effect of CHRM4 on BFU-E differentiation. Additionally, *GATA2* (136) and *ZFP36L2* (137), two key modulators of BFU-E differentiation, are established as direct target genes of the CHRM4/CREB pathway in BFU-Es (134, 135). As nerve fibers innervate the bone marrow, these findings collectively suggest that acetylcholine released from the nervous system crucially influences BFU-E differentiation via the CHRM4/CREB pathway.

### Targeting neuronal signaling in anemia.

Elevated acetylcholine levels have been reported in patients with MDS (138). Acetylcholinesterase, an enzyme responsible for the degradation of acetylcholine (133), is encoded by *AChE* located on Chr7q22. In MDS, deletion of Chr7q22 results in acetylcholine accumulation and triggers excessive cholinergic signaling. Deletion of *AChE* in primary human CD34^+^ hematopoietic stem and progenitor cells (HSPCs) is associated with defects in erythroid differentiation, whereas CHRM4 antagonists rescue these differentiation defects to restore erythropoiesis (139, 140). Furthermore, pharmacological inhibition of CHRM4 promotes BFU-E differentiation and alleviates anemia associated with MDS in genetically engineered *Mx1-Cre Srsf2^P95H/WT^* mice, correcting their BFU-E differentiation defect, reducing plasma EPO, and improving survival. In addition, targeted inhibition of CHRM4 shows benefits in mouse models of hemolysis and aging-associated anemia, suggesting translational therapeutic efficacy (134). Overall, these findings highlight CHRM4 signaling and early erythroid progenitor differentiation as attractive and emerging therapeutic targets for refractory anemia.

Since GPCRs constitute the largest class of pharmacologically druggable proteins, CHRM4 represents an attractive therapeutic target (141). To date, several antagonists such as PD102807 and PCS1055 have been developed that selectively target CHRM4, demonstrating high affinity and specificity in preclinical models (142). Pharmacodynamic and dose-tolerance studies have defined the minimal effective dose and highlighted CHRM4 as a potential therapeutic target in anemia (134). However, this approach is still at the preclinical stage, and more defined pharmacodynamic and pharmacokinetic optimization, along with reductions in potential nervous system toxicity, will be necessary to advance translation of CHRM4 antagonists into clinical development for ESA-resistant anemia therapy.

## Mitochondrial biogenesis and function in erythropoiesis

Mitochondria contribute to critical biological processes, including iron metabolism and mitophagy (143). Balance between antioxidant defense mechanisms and ROS generation is crucial for RBC survival, as prolonged ROS generation causes apoptosis, oxidative damage, and impaired erythropoiesis. During erythroid differentiation, lineage-specific progenitors exhibit increased mitochondrial energy production, respiration, and metabolic activity compared with HSPCs, driven by mTOR complex 1 (mTORC1), which promotes mitochondria-associated mRNA translation (144). In erythroid cells, mTORC1 signaling enhances translation of mitochondrial transcription factor A and prohibitin 2, both of which possess 5′ terminal oligopyrimidine (TOP) or TOP-like motifs, thereby promoting mitochondrial biogenesis, metabolic homeostasis, and terminal differentiation (144, 145). Loss of mitochondrial transcription factor A increases β-hydroxybutyrate synthesis, inhibiting histone deacetylases and impairing erythroid development through histone hyperacetylation and prolonged expression of HSPC-associated genes (146).

Mitochondrial clearance governed by receptor-mediated mitophagy is a complex mechanism during reticulocyte maturation in erythropoiesis (147). FoxO3 regulates autophagy-related genes, including NIX (also known as BNIP3L) and PTEN-induced kinase 1, thereby facilitating efficient mitochondrial removal (148, 149). NIX is a primary receptor that promotes autophagosome attachment to mitochondrial membranes, thereby facilitating mitochondrial clearance in developing RBCs. The diminished mitochondrial membrane potential observed in *Nix^–/–^* erythroid cells contributes to anemia and an increased number of immature reticulocytes, resulting from altered mitophagy during erythroid maturation (149, 150). Levels of EPO are increased in *Nix^–/–^* mice, suggesting a compensatory response to stimulate erythropoiesis. Together, these findings emphasize that precise control of mitochondrial biogenesis and mitochondrial clearance is imperative during erythropoiesis.

### Targeting mitochondrial pathways for therapeutic intervention.

Targeting mitochondrial dynamics and modulators is a promising approach to treating anemia. Various mitochondrial targets, including the Rieske iron-sulfur protein (RISP), autophagy-related gene (ATG5), Ran GTPase-activating protein 1 (RanGAP1), and pseudouridine synthase 1 (PUS1), regulate mitochondrial processes involved in RBC production by restoring homeostatic enucleation of RBCs (151–153) (Table 3). These mitochondrial modulators have substantial potential to enhance erythropoiesis by regulating metabolic pathways and alleviating oxidative stress, thereby improving anemic conditions. The small molecule mitapivat regulates the functional tetrameric form of the enzyme pyruvate kinase and enhances mitochondrial clearance, thereby promoting erythroid differentiation and erythropoiesis during anemia (154, 155). Furthermore, preclinical studies with sirolimus, an mTORC inhibitor, showed that it induces mitophagy and decreases ROS and erythrocytes containing mitochondria during terminal differentiation, thereby maintaining RBCs in SCD mice (156). In β-thalassemia, aberrant mTOR signaling limits Unc-51–like kinase 1 (ULK1) activity (157). ULK1, a serine/threonine protein kinase that functions as a key mediator of mitophagy, reduces α-globin toxicity in erythroid cells, making it an intriguing therapeutic target in β-thalassemia (158).

## Concluding remarks

Anemia is a global health concern, marked by disrupted erythropoiesis. The etiology of anemia encompasses ineffective erythropoiesis, genetic and splicing dysregulation, neuronal interactions, and mitochondrial dysfunction. Although many genetic and pharmacological therapies have been developed to date, challenges, such as EPO or ESA resistance, persist. Thus, anemia therapy requires a multimodal approach that includes altering hematopoietic signaling, correcting iron homeostasis, and targeting neuronal pathways. The development of small-molecule drugs and gene-editing strategies that specifically target the regulatory mechanisms of erythropoiesis may reduce off-target effects while stimulating RBCs. By integrating mechanistic insights into the genetic, splicing machinery, and neuronal regulation of erythropoiesis with clinical applications, the next generation of anemia therapies promises to be considerably more effective and precise. Such approaches may not only increase therapy effectiveness but also enhance responsiveness in patients with specific genetic or molecular profiles.

## Conflict of interest

OAW is a founder and scientific advisor of Codify Therapeutics. OAW has served as a consultant for Foundation Medicine Inc., Merck, Prelude Therapeutics, Amphista Therapeutics, MagnetBio, and Janssen and is on scientific advisory boards of Envisagenics Inc., Harmonic Discovery Inc., and Pfizer Boulder. LZ is an inventor on a patent on treating anemia with CHRM4 inhibitors (“Muscarinic acetylcholine receptor subtype 4 antagonists in the treatment of anemia”).

## Funding support

This work is the result of NIH funding, in whole or in part, and is subject to the NIH Public Access Policy. Through acceptance of this federal funding, the NIH has been given a right to make the work publicly available in PubMed Central.

- National Cancer Institute (NCI) MERIT Award 5R37CA276938 (to LZ).
- Congressionally Directed Medical Research Program Bone Marrow Failure Research Program Idea Development Award W81XWH2210380 (to LZ).
- Don Monti Memorial Research Foundation grant (to LZ).
- Cold Spring Harbor Laboratory (CSHL) President’s Council (to LZ).
- NCI Designated Cancer Center grant CA045508.
- Edward P. Evans Foundation grant (to OAW).
- Henry and Marilyn Taub Foundation grant (to OAW).
- Department of Defense Bone Marrow Failure Research Program grant W81XWH-12-1-0041 (to OAW).
- NIH/National Heart, Lung, and Blood Institute grant R01 HL128239 (to OAW).
- Leukemia and Lymphoma Society grant (to OAW).
- Pershing Square Sohn Cancer Research Alliance grant (to OAW).

## Figures and Tables

**Table 3:**
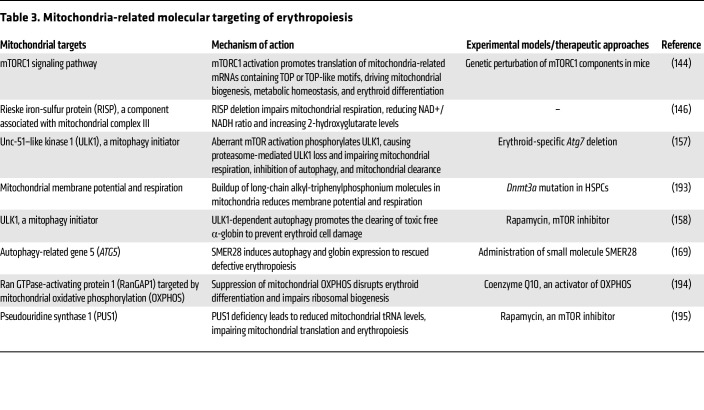
Mitochondria-related molecular targeting of erythropoiesis

**Table 2:**
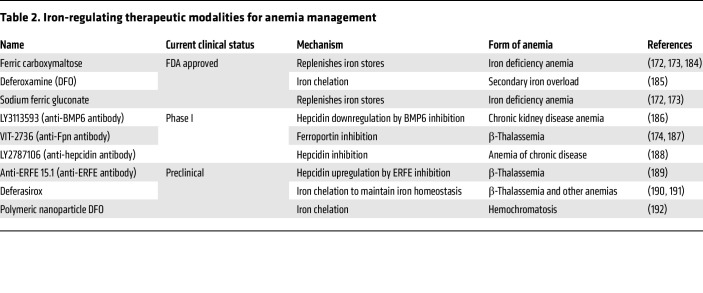
Iron-regulating therapeutic modalities for anemia management

**Table 1:**
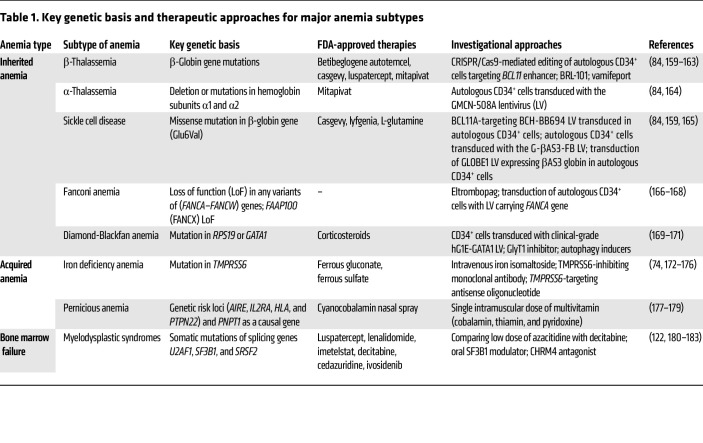
Key genetic basis and therapeutic approaches for major anemia subtypes

## Version 1 05/15/2026

Electronic publication
